# Rapid Photoinduced Self-Healing, Controllable Drug Release, Skin Adhesion Ability, and Mechanical Stability of Hydrogels Incorporating Linker-Modified Gold Nanoparticles and Nanogels

**DOI:** 10.1021/acsami.4c11908

**Published:** 2024-10-08

**Authors:** Samaneh Khodami, Mosayeb Gharakhloo, Serife Dagdelen, Piotr Fita, Jan Romanski, Marcin Karbarz, Zbigniew Stojek, Marcin Mackiewicz

**Affiliations:** † Biological and Chemical Research Center, University of Warsaw, Zwirki i Wigury 101, Warsaw 02-089, Poland; ‡ Faculty of Chemistry, University of Warsaw, Pasteura 1, Warsaw 02-093, Poland; § Institute of Experimental Physics, Faculty of Physics, University of Warsaw, Pasteura 5, Warsaw 02-093, Poland

**Keywords:** self-healing, NIR laser-beam treatment, drug release, gold nanoparticle, adhesiveness, nanogel

## Abstract

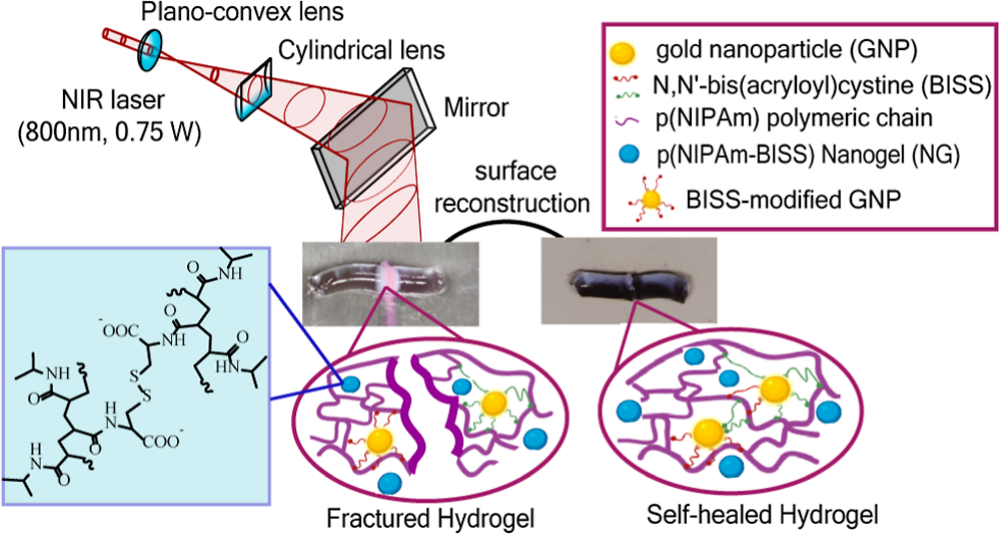

Appropriately modified thermoresponsive hydrogels, such as poly(*N*-isopropylacrylamide) hydrogels, bring an opportunity for a variety of biomedical applications. Incorporating compounds with different properties into poly(*N*-isopropylacrylamide) hydrogels offers opportunities to enhance their mechanical, self-healing ability, adhesiveness, thermal responsiveness, and drug release capabilities. In this study, we investigated the influence of Au–sulfur interactions on the properties of the poly(*N*-isopropylacrylamide) hydrogels after introducing *N,N′*-bis(acryloyl)cystine (a newly synthesized cross-linker), modified gold nanoparticles, and a p(NIPAm-BISS) nanogel into the hydrogel matrix. Our findings demonstrated that poly(*N*-isopropylacrylamide) hydrogels with these compounds exhibited higher mechanical strength (65% tensile stress and 25% elongation), faster thermal responsiveness, controllable self-healing [85% recovery after 2 min, using a NIR laser (800 nm, 0.75 W)], skin adhesiveness, and enhanced drug release (0.08 mg·mL^–1^, a 93% improvement). These results may contribute to advancements in the design of temperature-responsive hydrogels tailored for specific biomedical needs, such as targeted drug delivery with the use of a NIR laser and tissue engineering.

## 1 Introduction

Hydrogels are interesting materials that have found a broad range of applications and attracted interest in various disciplines, including engineering, materials science, and life sciences.^1−5^ Among them, the hydrogels based on poly(*N*-isopropylacrylamide) (pNIPAm) are extremely valuable for use in tissue engineering and drug delivery systems due to their thermoresponsive nature, where exact control over the swelling and shrinking processes can be used to get precise and controlled responses.^6^ Other properties, like self-healing, mechanical stability, and adhesiveness, can be achieved by formulating hybrid hydrogels based on the NIPAm monomer.^7,8^

One method used to enrich the properties of pNIPAm hydrogels is to introduce nanoparticles and produce nanocomposite hydrogels. There are many different nanoparticles, such as carbon-based, polymeric, and metallic nanoparticles, which can be combined with a polymeric network.^9−12^ These nanoparticles interact either chemically or physically with the network and provide hydrogels with specific characteristics. The first pNIPAm nanocomposite hydrogel was produced by Haraguchi and Takehisa, and the clay used in that nanocomposite improved significantly its mechanical strength and other characteristics.^13^

The self-healing ability of nanocomposite hydrogels is one of the important characteristics that has been extensively studied in the past decade for its potential to enhance gel functionality and durability.^14−16^ Gold nanoparticles (GNPs) are widely used as metallic nanoparticles in the development of nanocomposite hydrogels, particularly for enhancing the hydrogels’ properties, such as their self-healing ability.^17−19^ Due to their special optical, electrical, and catalytic properties, GNPs are valuable for improving the performance of hydrogels. Scientists can precisely tune the mechanical, optical, and biological characteristics of the resulting hybrid materials by adjusting the size, shape, and surface chemistry of GNPs.^20,21^ In addition, GNPs exhibit an intense absorption band in the near-infrared (NIR) spectrum. This phenomenon is caused by the collective oscillation of conduction band electrons induced by their interaction with an electromagnetic field. This phenomenon is known as localized surface plasmon resonance.^22^ The integration of a thermoresponsive polymer like pNIPAm with GNPs has the potential of creating nanocomposite hydrogels with advantageous synergistic features. These developments have resulted in the creation of stimulus-responsive hydrogels that can target therapeutic delivery and undergo quick regulated drug release from the gels.^23,24^ However, it is crucial to build a durable interaction between GNPs and the polymeric network. Marcelo et al. prepared smart catalyst nanocomposites based on pNIPAm and GNPs. In that hydrogel, the catechol groups were used to reduce HAuCl_4_ for functionalizing the hydrogels with GNPs.^25^ Also, the cross-linking copolymerization of pNIPAm and the monomers containing either thiol or dithiol groups was reported as an effective way to build Au-nanocomposite hydrogels.^26^ Consequently, it became evident that the Au–sulfur interactions in the hydrogels are able to recover damaged hybrid materials and provide benefits such as enhanced mechanical strength, biocompatibility, and adjustable optical characteristics.

**Scheme 1:**
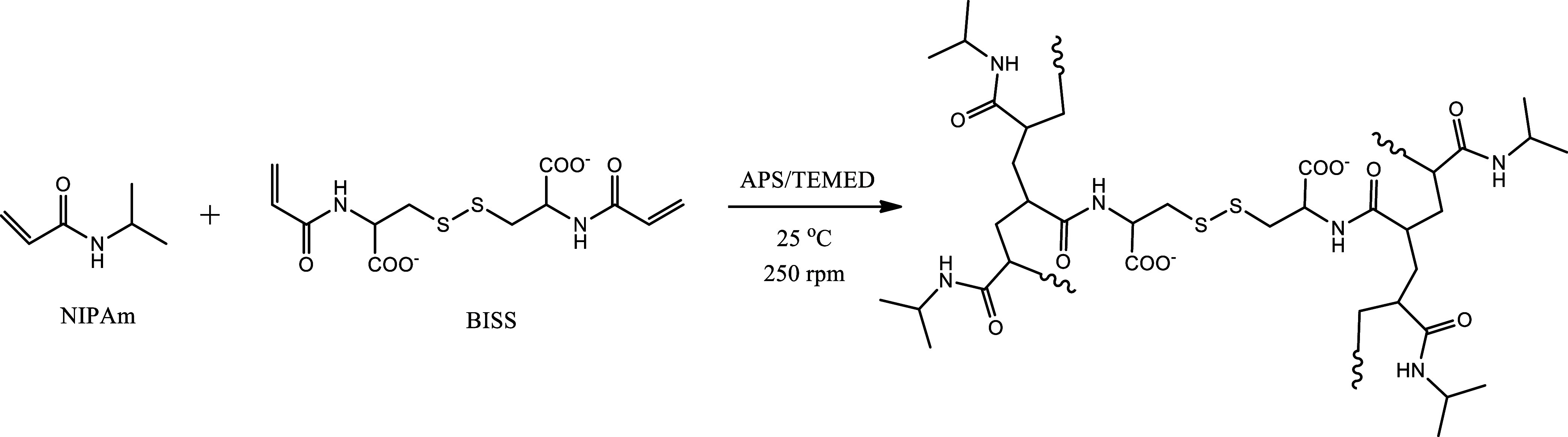
Scheme of Synthesis of p(NIPAm-BISS) Nanogel

Similarly, the integration of laser irradiation with the self-healing procedure should enable control over the repair mechanism. Significant progress in controlling and efficiently utilizing light-responsive components within the hydrogel matrix has been made in enhancing the self-healing action. This paved the way for the mechanisms that can accomplish on-demand repair.^27−30^ By using a NIR laser, the self-healing time of controlled hydrogels can be reduced to 1–2 min, resulting in a recovery rate of approximately 99%.^31^ Some varieties of self-healing hydrogels, however, take a longer time to repair the gel completely; for example, Chen et al.^32^ who produced a Cu-nanocomposite hydrogel and used an NIR laser to achieve self-healing after 10 min of radiation exposure. In addition to self-healing, Chen et al.^33^ designed an antibacterial gold nanocomposite hydrogel with real-time infection monitoring using a NIR laser, aimed at effective wound healing. These properties are particularly valuable in biomedical applications where precise and controlled drug release, such as the delivery of the anticancer drug doxorubicin (DOX), can be achieved.^34−36^ Wearable technology (including sensors and actuators), drug delivery systems, tissue engineering, and other fields could benefit greatly from these advanced features.^37−39^

Our paper presents research on hydrogels with a particular emphasis on the self-healing process initiated by laser technology, drug release capabilities, mechanical stability, and adhesive properties on a variety of substrates. We employed free-radical polymerization to prepare the nanocomposite hydrogel. Au nanoparticles were modified with a newly synthesized cross-linker containing –S–S– bonds, *N,N′*-bis(acryloyl)cystine (BISS). BISS-modified gold nanoparticles exhibited Au–sulfur interactions in the hydrogel network. These Au–sulfur interactions, upon NIR-laser irradiation, can impart self-healing properties and facilitate drug release within the hydrogel structure. The incorporation of the p(NIPAm-BISS) nanogel in the hydrogel exhibited greater temperature changes. Despite the low concentration of GNPs, the synthesized hydrogels exhibited a fast thermal response, rapid and controllable self-healing, and high drug release efficiency. In addition to enhancing self-healing and drug releasing, the hydrogels also exhibited strong adhesion to the skin. This adhesive property was achieved through the presence of excess carboxylic groups from BISS and other functional groups in the hydrogel, which created strong bonds between the hydrogel and the tissue. The adhesive properties of these hydrogels on various substrates have generated significant interest in biomedical applications, including tissue engineering scaffolds and wound dressings.^40−42^

Additionally, the mechanical strength of the gels was increased by the addition of GNPs due to the formation of a cross-linked network inside the gel matrix that could increase the elasticity and structural integrity of the material. With this advancement, the mechanically improved gels can be used in such fields as biomedical devices, biosensors, and actuators.^43−45^

In summary, by utilizing GNPs and Au–sulfur interactions, we successfully developed hydrogels with controllable self-healing and drug release capabilities along with strong mechanical strength and adhesion. These properties make the hydrogels promising candidates for further biomedical applications.

## 2 Materials and Methods

### 2.1 Chemicals and Materials

*N*-Isopropylacrylamide (NIPAm, >97.0%), potassium persulfate (KPS, >99.9%), ammonium persulfate (APS, >98.8%), *N,N,N′,N′-*tetramethylethylenediamine (TEMED, >99.0%), and tetrachloroauric (III) acid trihydrate (HAuCl_4_·3H_2_O, >99.9%) were purchased from Sigma-Aldrich. Citric acid monohydrate (pure p. a., C_6_H_8_O_7_·H_2_O) and sodium peroxide (NaOH, >98.8%) were bought from POCH. The *N,N′*-bis(acryloyl)cystine (BISS) cross-linker used in this study, was synthesized by us according to our previous publication.^46^ All chemical compounds were used without alteration except for the NIPAm monomer, which was recrystallized from a toluene/hexane mixture (30:70 v/v). For the preparation of the solutions, high-purity water of a 0.05 μS·cm^–1^ conductivity was prepared by using a Milli-Q Plus/Millipore purification system.

### 2.2 Synthesis of Gold Nanoparticles

Gold nanoparticles (GNPs) were synthesized following the established procedures with slight modifications.^47^ Briefly, either 125 μL or 375 μL or 625 μL of a 0.12 M solution of HAuCl_4_ was mixed with 50 mL of distilled water and gently brought to boiling. Subsequently, either 1 mL or 3 mL or 5 mL of a 38.7 mM solution of citric acid, with pH adjusted to 6–7 using NaOH, were added incrementally under continuous stirring until the solution turned deep red, which indicated GNP formation. By maintaining the ratios of HAuCl_4_ and citric acid, the solutions of various GNP concentrations were obtained and entitled GNP1, GNP2, and GNP3. Finally, the solutions were cooled to room temperature, purified by dialysis, and stored in light-protected containers.

### 2.3 Synthesis of Nanogels

P(NIPAm-BISS) nanogels (NG) were synthesized through a semibatch surfactant-free precipitation polymerization method [nanohydrogel with *N,N*^*’*^-bis(acryloyl)cystine cross-linker for high drug loading] (Scheme 1). The polymerization took place in a round-bottom, two-neck, 50 mL flask equipped with a stirrer, operating at approximately 250 rpm, and inlet and outlet ports for inert gas. The primary monomer, NIPAm (97 mM), was dissolved in a flask with 15 mL of deionized water, along with TEMED (17 μL) and APS (2.3 mM). After a 30 min deoxygenation period at 25 °C, the dropwise addition of an aqueous solution containing the cross-linker BISS (3 mM, in 2 mL of deionized water) was conducted using a peristaltic pump with a flow rate of 2.6 mL·h^–1^. The reaction proceeded for 2 h. After that, the obtained mixture underwent dialysis against deionized water for 1 week using a dialysis tube with a cutoff molecular weight of 10,000 Da (Spectra/Por 7 Dialysis Membrane) to remove any unreacted reagents. The resultant nanogel solution had a concentration of ca. 5 mg·mL^–1^ and an average size of approximately 26 ± 6 nm [based on transmission electron microscopy (TEM) results]. The TEM image, the graph of the size distribution of the nanogel particles (determined using Nano Measurer 1.2 software based on TEM images), and the dynamic light scattering (DLS) graph are given in the Supporting Information (Figures S2 and S3).

### 2.4 Synthesis of Nanocomposite Hydrogels

For the synthesis of nanocomposite hydrogels, free-radical polymerization was employed. Various concentrations of NIPAm (1.00 and 1.40 M), BISS (0.35, 1.00, and 2.00 mM), GNP (GNP1, GNP2, and GNP3), KPS (11.00 mM), and TEMED (2.65 mM) solutions were used. The polymerization process comprised three distinct steps. Initially, BISS and 2.5 mL of GNP solution were mixed and sonicated for 20 min in a sonication bath. Meanwhile, the NIPAm monomer was dissolved in either 2.5 mL of water or NG dispersion in an ice bath under a nitrogen atmosphere. Subsequently, in the second step, the BISS-modified GNP solution was introduced to the monomer solution, and the resulting mixture was stirred in an ice bath under an argon (Ar) environment for 20 min. In the final step, the KPS solution and TEMED were swiftly added. The polymerization commenced upon the introduction of KPS and proceeded overnight in a refrigerator.

### 2.5 UV–Vis Spectroscopy

To confirm the functionalization of GNPs, changes in optical transmittance were examined at a wavelength of 550 nm utilizing a Lambda 25, PerkinElmer UV–vis spectrometer.

### 2.6 Dynamic Light Scattering

The hydrodynamic diameters of the GNPs and the mixture of GNPs and BISS were evaluated utilizing a Malvern Zetasizer apparatus (Nano ZS, UK). This instrument featured a 4 mW helium–neon laser that emitted light with a wavelength of 632.8 nm. The measurements were conducted at a scattering angle of 173°.

### 2.7 Morphology

TEM investigations were performed with a Talos F200X (FEI Company) microscope operated at 200 kV to characterize the NG particles, GNPs, BISS-modified GNPs, and gel samples. The measurements were performed in both TEM and scanning TEM (STEM) modes using a high-angle annular dark-field (HAADF) detector and energy-dispersive X-ray spectroscopy mode of a Bruker BD4 spectrometer to evaluate the elemental distribution of carbon, sulfur, and gold.

The morphology and elemental composition of the gel sample were investigated with a Quantax 400 Bruker EDS/energy-dispersive X-ray (EDX) detector in conjunction with a Merlin, ZEISS field emission scanning electron microscope (FESEM). Prior to measurement, a Mini Sputter Coater (Polaron SC7620) was used to deposit a 5 nm layer of Pd alloy onto the samples under vacuum. The samples were flash-frozen in liquid nitrogen and then freeze-dried using a specialized lyophilizer, Labconco FreeZone device at −82 °C and a pressure of 0.03 mbar.

### 2.8 Mechanical Performance

The tensile properties of the hydrogels were assessed by utilizing an EZ-SX Shim-Pol tensile test machine both before and after performing the self-healing. All experiments were conducted in triplicate. Tensile tests were executed at a constant strain rate of 50 mm·min^–1^.

The compression tests were conducted using a 20 N load cell. Cylindrical hydrogel samples, measuring 10 mm in diameter and 10 mm in height, underwent compression at a rate of 50 mm·min^–1^ under ambient conditions.

Rheological assessments were performed using an MCR302 strain-controlled rheometer (Anton Paar, Graz, Austria) with plate-and-plate geometry (diameter, 15 mm). Two distinct modes were employed: (a) an amplitude sweep test was conducted at a fixed angular frequency of 10 rad·s^–1^ at 20 °C, where the amplitude of oscillatory strains ranged from 0.01 to 1000%. Additionally, frequency sweep tests were conducted with a fixed strain amplitude of 10% across a frequency range of 1–100 rad·s^–1^. A PolyScience circulating bath was used to control the temperature during the rheological experiments. To minimize evaporation and maintain the temperature constant, a cap was employed throughout all measurements. The measurements were carried out in triplicate.

### 2.9 Self-Healing Tests and Photothermal Experiments

**Figure 1:**
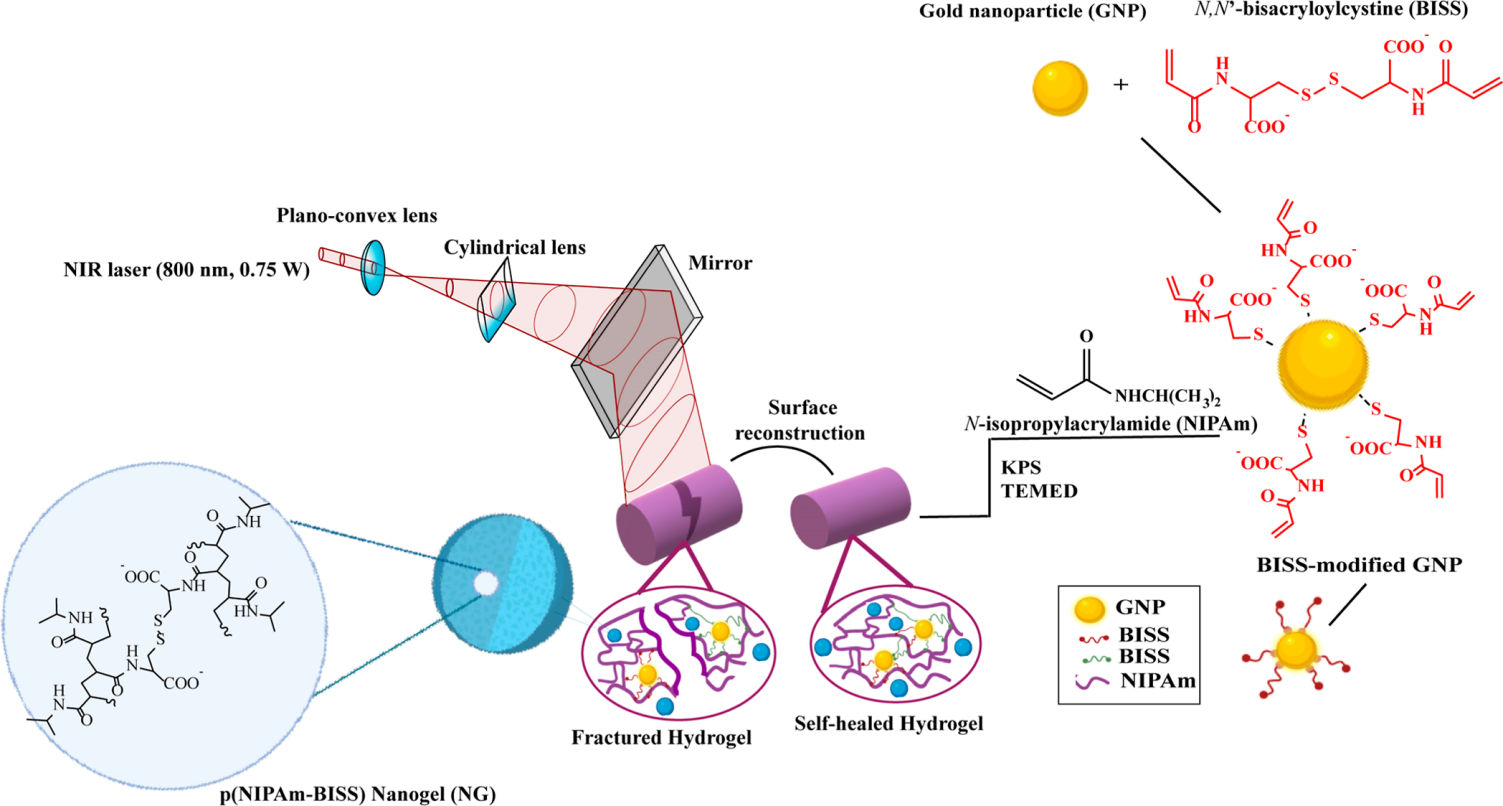
Scheme of polymerization of the nanocomposite hydrogel incorporating BISS-modified GNPs and NG and sample irradiation by the NIR laser (800 nm) to regenerate Au–sulfur bonds and facilitate the healing process of the fractured part of the hydrogel.

For self-healing tests, the hydrogel samples in the form of a cylinder of diameter ca. 5 mm were cut in half with a sharp blade. The resulting halves were placed on a glass slide and brought into contact at the position of the cut. The joint was irradiated from above with laser light. A Ti:sapphire femtosecond laser (Spectra Physics MaiTai) tuned to a central wavelength of 800 nm was employed. Its output beam was shaped with the help of a spherical and a cylindrical lens to form an elliptical spot at the position of the sample (see Figure 1). The minor and major axes of the ellipse had lengths of approximately 3 and 8 mm (measured at half-maximum intensity), respectively. The longer axis was parallel to the cut. The power of the laser beam was reduced to 0.75 W, which resulted in light intensity at the sample position of ca. 4 W·cm^–2^. The experiment was conducted at room temperature. The self-healing efficiency was studied by doing the tensile tests.

In the studies of the hydrogel photothermal response, the same cylindrical gel geometry was employed. The thermal images of the samples were obtained with an infrared thermal imaging camera (Fluke Ti400).

### 2.10 Adhesiveness Test

#### 2.10.1 Lap-Shear Test

Lap-shear testing was carried out by using the tensile machine (EZ-SX Shim-pol). The adhesive strength values were calculated by dividing the maximum shear force by the standardized contact area of 200 mm^2^, achieved by overlapping tissue samples. Each hydrogel specimen underwent testing with three replicates. Fresh commercially available vacuum-sealed porcine tissue was stored temporarily at 4 °C until use. Rectangular sections measuring 10 mm × 100 mm were precisely cut from the fresh porcine using a razor blade. Excess fat was removed to ensure uniformity. The thickness of the tissue sections was 2 mm.^48^

To preserve tissue hydration, the tissue was immersed in a buffer solution prior to testing. Preloading of the tissue–gel–tissue piece was applied at 1.5 N in the normal direction for a duration of 10 s. The overlapping area was 10 × 20 mm. A 20 N load cell, integrated with the tensile machine, measured the lap shear of the tissue–gel–tissue stickiness. The samples were clamped between two fixtures and subjected to parallel pulling at a velocity of 50 mm·min^–1^.

#### 2.10.2 T-Peel Test

Using the aforementioned tensile machine, T-peel tests were performed. This entailed bonding two porcine samples, each 10 mm wide, 100 mm long, and 2 mm thick, using the hydrogel. The overlapping region of the tissue samples was sized 10 mm × 20 mm. Following this, the free ends of both tissue samples were individually secured to the tensile machine grips.

Subsequently, the upper tissue was subjected to upward pulling at a peeling angle of 180° and a constant velocity of 50 mm·min^–1^, while the peeling force (*F*_p_) was simultaneously recorded. The measurement of the work of adhesion, necessary for the separation of the hydrogel-bonded tissues, was subsequently calculated as 2 × *F*_p_·*w*^–1^, where *w* is the width of the sample.^49^

### 2.11 Determination of Load and Release of Doxorubicin (DOX)

#### 2.11.1 Loading of DOX into the Hydrogels

DOX, a widely used anticancer drug, was incorporated into the hydrogels by using the incubation method. Initially, 10 mg of the hydrogels was combined with 0.5 mg of DOX and dispersed in 10 mL of a phosphate buffer solution (pH = 7.4; 0.15 M). The mixture underwent heating process at 45 °C for 10 min, followed by cooling to 25 °C. This heating and cooling cycle was repeated several times to ensure thorough incorporation. Subsequently, the supernatant containing any unbound DOX was replaced with fresh phosphate buffer solution, and this process was iterated until excess DOX was effectively removed. The amount of DOX loaded into the gels was determined by subtracting the mass of free DOX present in the supernatant from the total mass of DOX utilized in the loading process (eq 1). The concentration of DOX in the solution was quantified by measuring its absorbance at 480 nm. A calibration plot was employed for accurate determination.^50^

Drug loading capacity (DLC) was evaluated using eq 1
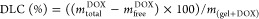
1where *m*_total_^DOX^ is the total mass of DOX used in the loading process, *m*_free_^DOX^ is the mass of DOX that was not bound to the gel, and *m*_(gel + DOX)_ is the mass of the gel with DOX.

#### 2.11.2 DOX Release from the Hydrogels

The release of DOX from the hydrogels was assessed after laser irradiation by using UV/vis absorption spectroscopy. The experimental setup mirrored that of the self-healing tests with the exception of removing the cylindrical lens. Consequently, the laser spot on the sample became circular and measured ca. 8 mm in diameter (at half-maximum). The laser beam operated at 0.75 W, yielding a light intensity of approximately 4 W·cm^–2^. A solution consisting of 10 mL of phosphate buffer containing the DOX-loaded gel was prepared. Then the absorbance of DOX released into the supernatant was measured.^51^

## 3 Results and Discussion

### 3.1 Preparation of the Hydrogel

This study aimed at developing a hydrogel with several properties, including self-healing, self-adhesiveness, drug release capabilities, and mechanical stability by incorporating Au–sulfur interactions into a pNIPAm hydrogel network. To achieve this, gold nanoparticles (GNPs) and an *N,N′*-bis(acryloyl)cystine (BISS) cross-linker were synthesized and integrated with the pNIPAm network through the Au–sulfur bonding. The hydrogels were synthesized by using the free-radical polymerization method. Within the hydrogel structure, the synergistic interactions between GNPs and the sulfur-containing polymer chains contributed to the self-healing property and mechanical stability. The optothermal characteristics of GNPs enabled reversible switching of Au–S interactions using an NIR laser. These reversible interactions resulted in the reconstruction of polymer networks in the damaged areas, see Figure 1. In parallel, BISS and pNIPAm were engaged in the formation of covalent cross-linking, which enhanced the mechanical properties. Various hydrogel formulations of different concentrations of the NIPAm monomer, BISS, and GNPs were synthesized. Extra formulations incorporating a p(NIPAm-BISS) nanogel (NG) were prepared as well. Although we synthesized and examined the self-healing properties of all formulations with different concentrations, only the hydrogels listed in Table 1 demonstrated self-healing properties following NIR laser irradiation, and these formulations were selected for additional assessment. The remaining formulations were not mentioned because they lacked the necessary self-healing properties.

**Table 1:** Concentrations of Substrates for Hydrogel Synthesis

hydrogel	NIPAm/M	BISS/mM	AuNP^a^	NG suspension/mL	water/mL
(pNGB1)	1.0	2.00	GNP2		2.5
(pNGB2)	1.4	0.35	GNP3		2.5
(pNGB3)	1.0	1.00	GNP3		2.5
(pNGB4)	1.4	1.00	GNP3		2.5
(pNGB_NG1)	1.0	1.00	GNP3	2.5	
(pNGB_NG2)	1.4	0.35	GNP3	2.5	
(pNGB_NG3)	1.4	1.00	GNP3	2.5	

a In case of AuNPs, go to the Synthesis of Gold Nanoparticles section.

### 3.2 Dynamic Light Scattering and UV–Vis Spectroscopy

**Figure 2:**
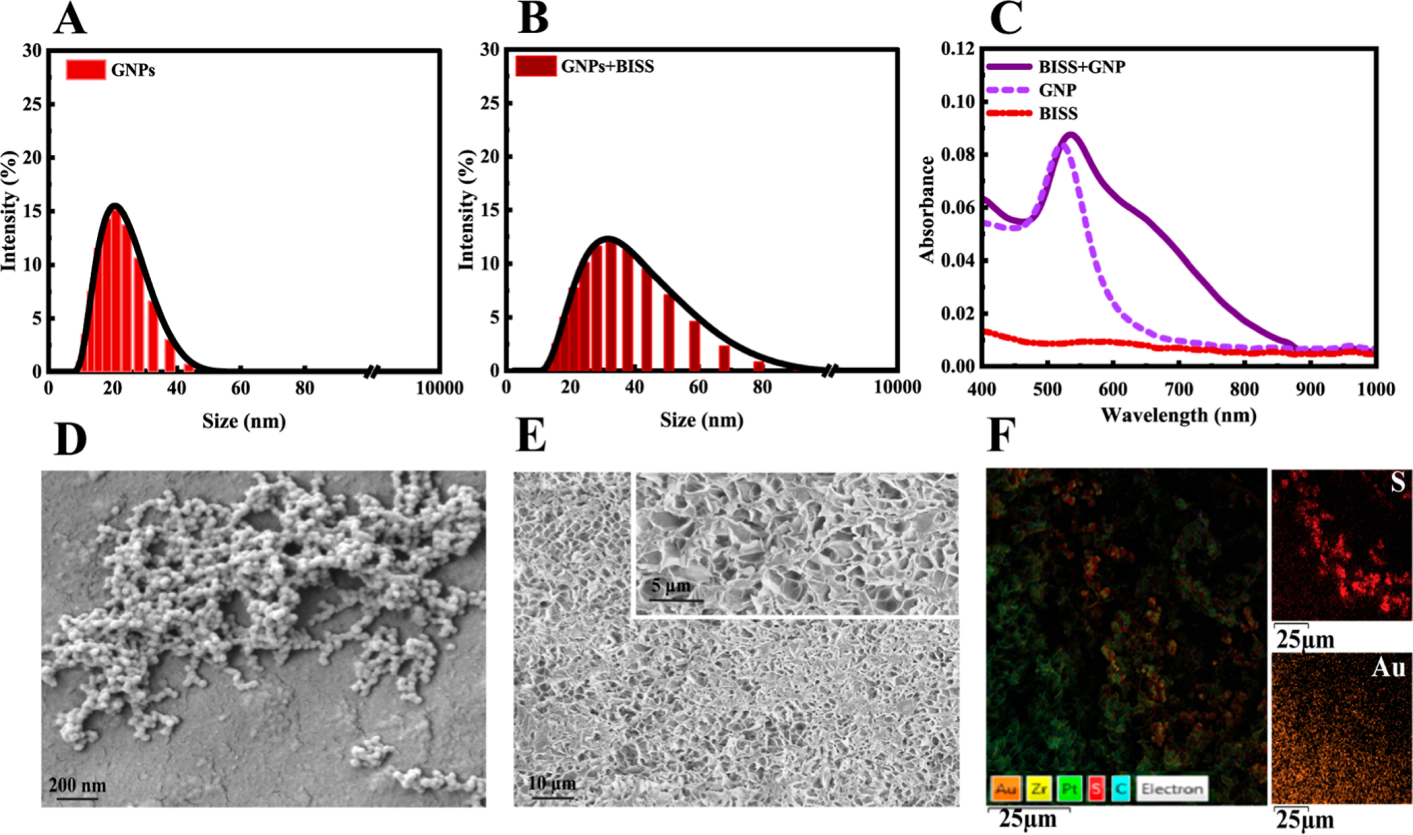
Size distribution of synthesized GNPs (A), size distribution of BISS-modified GNPs (B), UV–visible spectra of GNPs, BISS, and BISS-modified GNPs (C), SEM image illustrating the morphology of GNPs (D), SEM images depicting a cross-section of the hydrogel (E), and distributions of Au and sulfur within the hydrogel captured by EDX-SEM (F).

Dynamic light scattering (DLS) analysis was carried out to examine the size distribution of GNPs (Figure 2A). The results indicated the presence of GNPs with an average size of 22 ± 8 nm. The average hydrodynamic diameter, derived from the DLS data, served as a vital parameter for characterizing GNPs within the hydrogel matrix. Moreover, the relatively narrow distribution suggested consistent material properties and performance. The DLS analysis confirmed the successful synthesis of GNPs.

To produce the hydrogels, we initially synthesized GNPs dispersed in water. Following this, we introduced specific quantities of BISS, which contains –S–S– group within its structure. The size of the mixture of BISS-modified GNPs was 33 ± 15 nm, with a wider distribution but still within a good range (see Figure 2B). Also, the BISS molecules underwent targeted adsorption onto the surface of the GNPs, forming Au–sulfur bonds. An analysis of the UV–vis absorption spectra showed a distinctive purple peak shifting from 551 to 570 nm upon the addition of BISS (see Figure 2C). This indicated the successful functionalization of GNPs.^52^

### 3.3 Surface Microscopy

The structure and morphology of GNPs and the hydrogel were investigated using scanning electron microscopy (SEM).

In Figure 2D, an SEM image of GNPs is shown. The image illustrated the particle size and dispersity of the GNPs after drying. The measured mean diameter of the dried particles was 25.3 ± 9.1 nm, indicating a uniform shape and dispersion. This finding agreed with the results obtained from DLS analysis. Typical TEM images of GNPs with and without BISS, and EDS-HAADF elemental mapping of Au and sulfur in these solutions, are added to the Supporting Information (Figure S5).

To visualize the three-dimensional network of the hydrogels, the samples were freeze-dried and fractured.^53^Figure 2E presents representative images of the cross-sectional surface. The hydrogel displayed a honeycomb-like structure with a uniform mesh, indicating a homogeneous structure and morphology. The well-defined network architecture of the hydrogel serves as a crucial feature that significantly influences other properties. As depicted in Figure 2F, the EDX analysis demonstrates good dispersion of sulfur and Au atoms within the hydrogel matrix. TEM images of the hydrogel and EDS-HAADF elemental mapping of Au, sulfur, and carbon within the hydrogel have been added to the Supporting Information (Figure S4). It is clearly visible that the GNPs are uniformly distributed within the polymer matrix.

### 3.4 Temperature Responsiveness of the Hydrogels

The thermal behaviors of the hydrogels of various ratios of NIPAm, GNPs, BISS, and NG (listed in Table 1) were examined. The hydrogels were exposed to laser irradiation (800 nm, 0.75 W), and their thermal changes over time were monitored by using a thermal imaging infrared camera.

**Figure 3:**
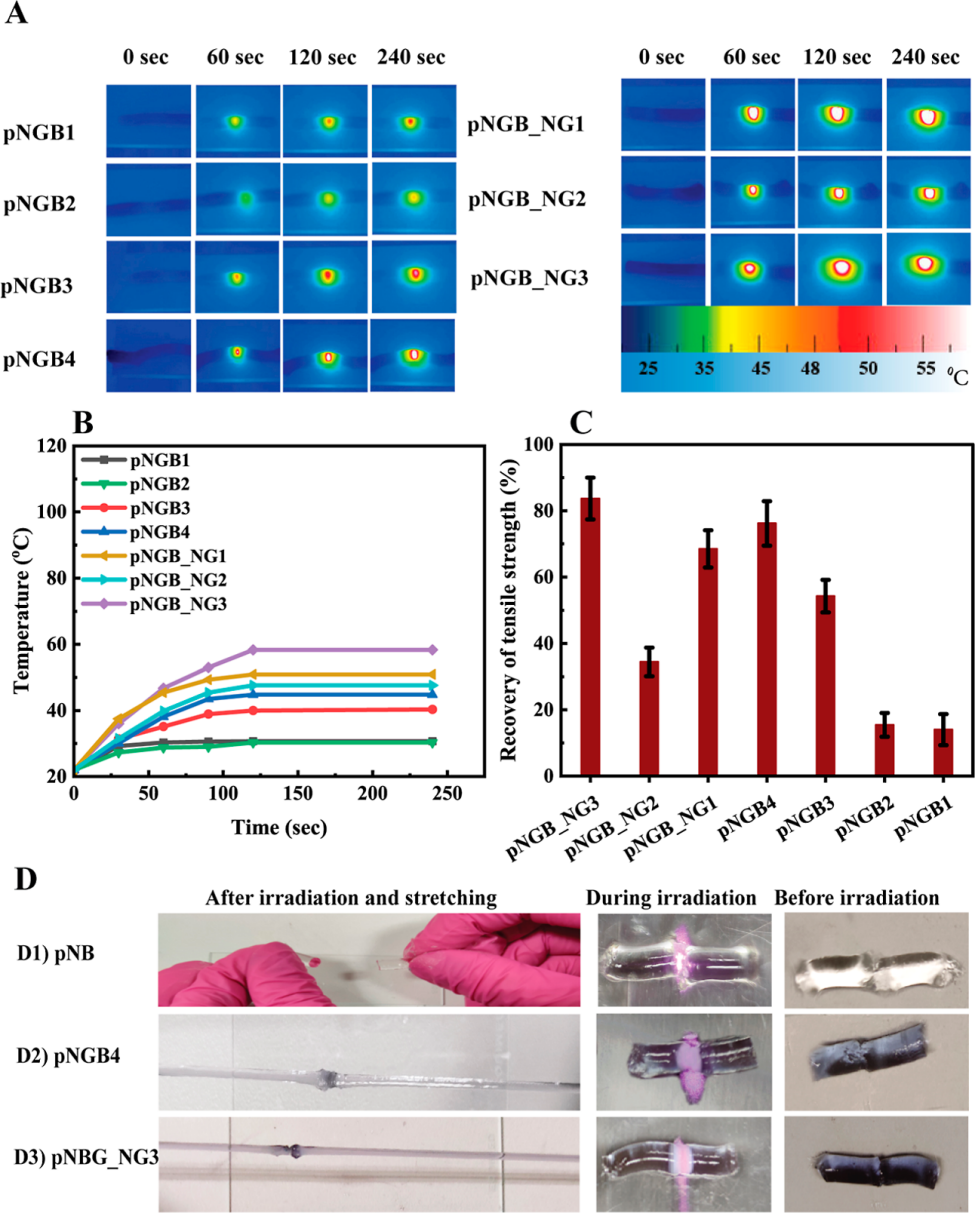
Temperature mapping (A) and temperature changes of the prepared hydrogels over time of exposure (B), recovery of tensile strength in the prepared hydrogels following the self-healing process facilitated by the NIR laser irradiation (the mean values for three replicate samples ± standard deviation are shown) (C), and self-healing process photography before, during, and after laser irradiation (D) for pNB (D1), pNGB4 (D2), and pNGB_NG3 (D3).

The results shown in Figure 3A indicate that the composition of the hydrogels significantly influenced the thermal behavior. Hydrogels with higher amounts of GNPs and BISS exhibited faster and more pronounced thermal changes compared to those with lower amounts. The volume phase transition of hydrogels occurred more quickly, and the time necessary for maximum shrinkage was shortened due to the higher Au content. Additionally, hydrogels containing the NG dispersion showed enhanced temperature responsiveness. Introduction of NG reduced the mechanical rigidity of the hydrogel as seen in the tensile test results. The use of NG could increase the intermolecular spacing between polymeric chains and reduce the number of cross-linking spots between the chains. In general, the nanogels can alter the water content and reduce the mechanical strength. These factors resulted in enhanced temperature-responsive changes and improved thermal conductivity. Also, the larger concentration of NIPAm in pNGB-NG1 compared to pNGB3 is expected to result in an increased ability of the hydrogel structure to absorb and retain heat. This could lead to an increase in temperature following laser irradiation, particularly if the hydrogel efficiently absorbed the irradiation energy.^54,55^

Next, the temperature changes during laser irradiation were monitored over time. Figure 3B illustrates the temperature changes observed during 250 s of laser irradiation. All samples exhibited a rapid increase in temperature, followed by a plateau. The hydrogels with higher amounts of GNPs and BISS showed faster temperature increases and reached 42 °C after 120 s, surpassing the phase transition temperature of pNIPAm. Conversely, the hydrogels with lower amounts of GNPs and BISS did not reach the phase transition temperature of pNIPAm. After incorporating NG into the hydrogels, they showed a rapid temperature increase, and for pNGB-NG3, the temperature reached 60 °C after 120 s.

### 3.5 Self-Healing Investigation

To investigate the self-healing behavior induced by irradiation, hydrogel samples were cut and exposed to the NIR laser beam (800 nm) for 2 min, as depicted in Figure 3D. The light transmittance at the fracture site decreased after irradiation; this was accompanied by a color change to opaque white, indicating that the temperature exceeded the critical phase transition temperature of pNIPAm. Subsequently, the hydrogel exhibited healing and regained its original stretchable form. In contrast, the pure pNIPAm hydrogel lacked self-healing ability, as evidenced in Figure 3D1. Notably, fractures in pure hydrogel samples remained unrepaired after NIR laser irradiation, highlighting the role of laser light interaction with the surface plasmon of GNPs and the restoration of Au–sulfur bonds in the healing process. As depicted in Figure 3D2,D3, both GNP-cross-linked hydrogels were cut into separate pieces and subsequently brought into contact. Upon exposure to the NIR laser beam, the pieces underwent self-healing within 2 min, and the healed GNP-cross-linked hydrogels regained considerable stretch.

Additionally, the recovery of tensile strength (in %) for all prepared hydrogels (see Table 1) after treatment/irradiation with laser light is depicted in Figure 3C. These tensile experiments indicate the correlation between healing efficiency and hydrogel composition.

Results indicated that the recovery was enhanced with increasing concentrations of BISS and GNPs in the hydrogel. Minimal opaque regions observed at the fractures of the pure pNIPAm hydrogel suggested that while the temperature could approach the critical phase transition temperature of pNIPAm, it failed to induce a full phase transition. Moreover, low concentrations of GNPs or BISS were insufficient to meet the temperature change required for the self-healing process. The samples with lower concentrations exhibited limited post-healing stretchability, emphasizing the importance of the presence of GNPs for enhancing the self-healing ability.

Also, the elevated concentrations of NIPAm monomer and BISS increased the total number of cross-linking points within the gel networks, further enhancing mechanical integrity. According to the literature, the thermo-sensitivity of pNIPAm is essential for NIR laser-induced self-healing, which provides remote control capabilities.^56^

Consequently, pNGB_NG3 and pNGB4 were selected as optimal samples for further investigation because of their greater recovery percentage (pNGB_NG3 and pNGB4 regained 84% and 76% of their original strength after self-healing). However, since the inclusion of NG reduces strength, a comprehensive examination of other gel properties was conducted and is reported in subsequent sections.

### 3.6 Mechanical Properties

#### 3.6.1 Tensile Test

**Figure 4:**
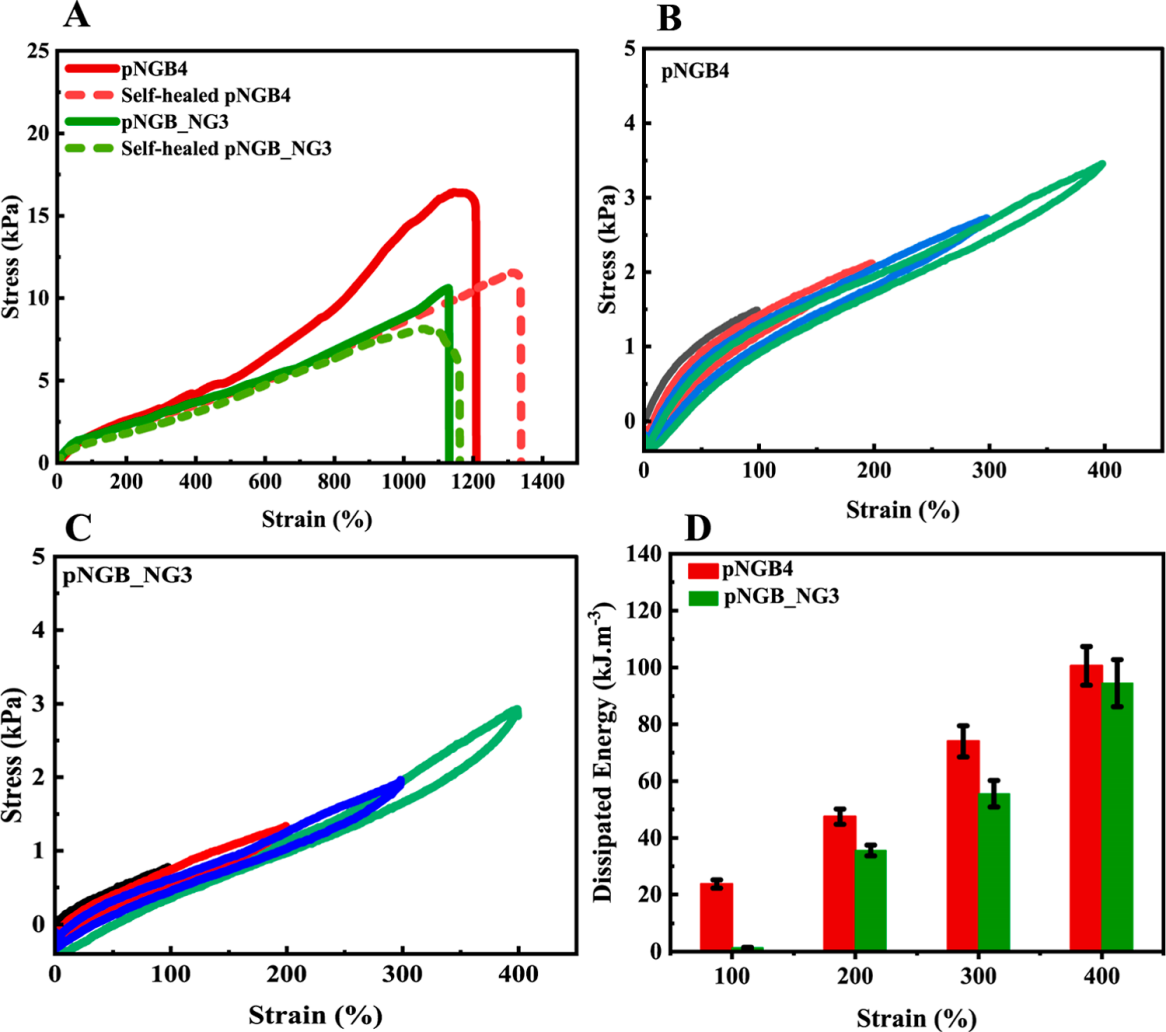
Tensile curves for pNGB_NG3 and pNGB4 before and after self-healing (A), cyclic tensile curves of pNGB4 (B) and pNGB_NG3 (C) under different strains: 100% (black line), 200% (pink line), 300% (blue line), and 400% (green line). Dissipated energy bar chart for pNGB_NG3 and pNGB4 (the mean values for three replicate samples ± standard deviation are shown) (D).

Figure 4 presents the mechanical properties of pNGB4 and pNGB_NG3 hydrogels determined through typical and cyclic tensile tests with optimal gel formulations.

Figure 4A depicts typical stress–strain curves of the pNGB4 and pNGB_NG3 hydrogels. These curves provide insights into the mechanical behaviors of the hydrogels before and after self-healing. The results indicated that pNGB4 hydrogels can be stretched to 1200% elongation compared to their initial length and ruptured at a stress of 17 kPa. The hydrogel showed self-healing capabilities after laser irradiation, achieving 1400% elongation and withstanding a stress at break of 11 kPa, which indicates the formation of new Au–sulfur bonds during the self-healing process.

Similarly, pNGB_NG3 hydrogels exhibited high stretchability, reaching 1110% elongation compared to the initial length and rupturing at a stress of 11 kPa. After self-healing, the hydrogel showed approximately 1180% elongation and broke at a stress of 8 kPa, indicating effective self-repair mechanisms involving rearrangement and formation of new Au–sulfur bonds.

The obtained results demonstrated the significant influence of Au–sulfur interactions on the mechanical properties and self-healing capabilities of the prepared hydrogels. Laser irradiation promoted and facilitated self-healing through the formation of new Au–sulfur bonds. The stress–strain curves of pure hydrogels (1.4 M NIPAm, 0.9 mM BISS) with and without NG have been added to the Supporting Information (Figure S1). These results show that the addition of GNPs can increase the mechanical stability.

Figure 4B,C presents representative cyclic tensile tests on the pNGB4 and pNGB_NG3 hydrogels, respectively. Loading–unloading tests were conducted to evaluate the energy dissipation of the hydrogels (Figure 4D). The applied strain started at 100% and was progressively increased by 100% after each cycle until reaching 400%. The behavior of the hydrogel composites during cyclic loading and unloading was interpreted based on the observed hysteresis loops, which indicated the dissipation of energy during the mechanical testing.

The obtained results highlight the capability of pNGB4 and pNGB_NG3 hydrogels to effectively dissipate mechanical energy during cyclic loading–unloading tests. The incorporation of the nanogel into the hydrogel matrix altered its mechanical properties, resulting in softer behavior and reduced energy dissipation. However, these changes are minor and negligible. In general, nanogels can alter the water content and act as defects or stress concentrators under stress, reducing the mechanical strength. However, the small differences observed in Figure 4 indicate that the overall cross-linking density remains unaffected due to the good homogeneity and strong interfacial interaction between the p(NIPAm-BISS) nanogel and the hydrogel structure.

These findings contribute to the understanding of the mechanical behavior and energy dissipation mechanisms of these hydrogel composites, which are essential for various applications requiring robust and relatively tough materials.

#### 3.6.2 Compression Test

**Figure 5:**
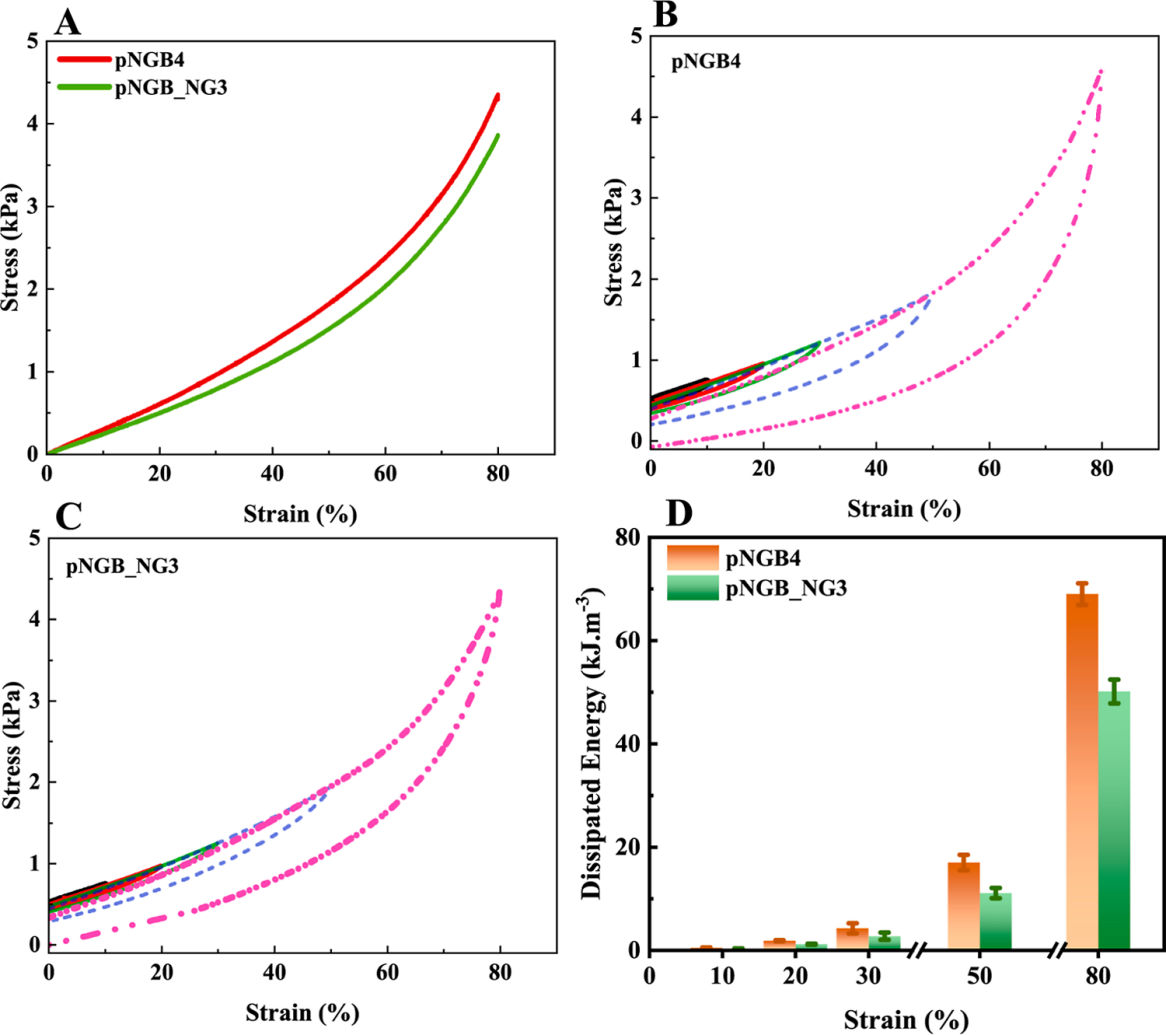
Normal compression curves for pNGB_NG3 and pNGB4 (A), cyclic compression curves of pNGB4 (B), and pNGB_NG3 (C) under different strains: 10% (black), 20% (red), 30% (green), 50% (blue), and 80% (pink). Dissipated energy graph for the optimal composition of pNGB_NG3 and pNGB4 (the mean values for three replicate samples ± standard deviation are shown) (D).

To further evaluate the compression recovery performance, the hydrogels underwent typical and cyclic compression tests with increasing strain (10–80%), as depicted in Figure 5.

Figure 5A displays the compression test results for the pNGB4 and pNGB_NG3 hydrogels. The results indicated that pNGB_NG3 was slightly softer than pNGB4, which is consistent with the findings from the tensile tests.

The loading–unloading compression measurements were aligned with the cyclic loading–unloading tensile tests. As depicted in Figure 5B,C, both pNGB4 and pNGB_NG3 hydrogels exhibited compression unloading curves that mirrored the loading curve up to 30% deformation, with small hysteresis loops, confirming the tensile results. Larger hysteresis loops were observed at higher compressions, particularly at 80%. Additionally, Figure 5D reveals that pNGB4 exhibited higher dissipated energy than pNGB_NG3.

Based on the above results, the prepared hydrogels not only exhibited enhanced compressive strength but also good dissipated energy during cyclic compressive loading–unloading.

#### 3.6.3 Rheology Test

**Figure 6:**
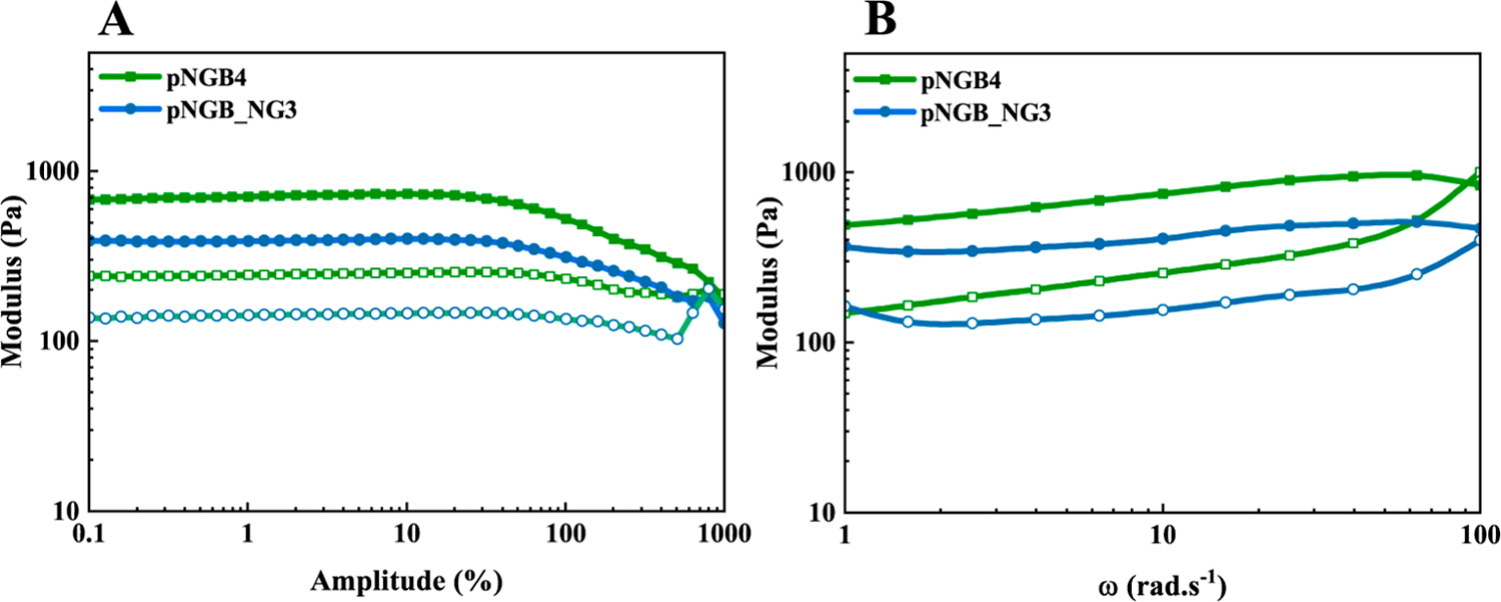
Storage (filled symbols) and loss modulus (empty symbols) plotted versus amplitude (A) and angular frequency (B) for pNGB_NG3 and pNGB4. The tests were done on three replicate samples, and the mean values are depicted in the graph.

Figure 6 presents the storage modulus (*G*′) and loss modulus (*G*″) of the hydrogels as functions of amplitude and frequency. Figure 6A displays the storage modulus (*G*′) and loss modulus (*G*″) of the pNGB4 and pNGB_NG3 hydrogels as functions of amplitude (γ) to determine the linear viscoelastic region of the hydrogel. Both *G*′ values are higher than *G*″, which indicates their gel-like properties.

Figure 6B presents the findings of the frequency sweep tests. A γ value of 10% was selected for the frequency sweep measurements. The results revealed that *G*′ exceeded *G*″ across the entire angular frequency (ω) range, indicating predominant viscoelastic behavior. The variation of *G*′ and *G*″ with angular frequencies illustrates typical behavior for gel-like samples, with both moduli exhibiting little change with frequency. The highest storage modulus was observed for pNGB4 at *G*′ ∼ 960 Pa, whereas for pNGB_NG3, *G*′ was ca. 510 Pa. These results indicated that the pNGB4 hydrogel exhibited higher stiffness compared to pNGB_NG3; it was consistent with other mechanical tests.

### 3.7 Adhesion Properties

**Figure 7:**
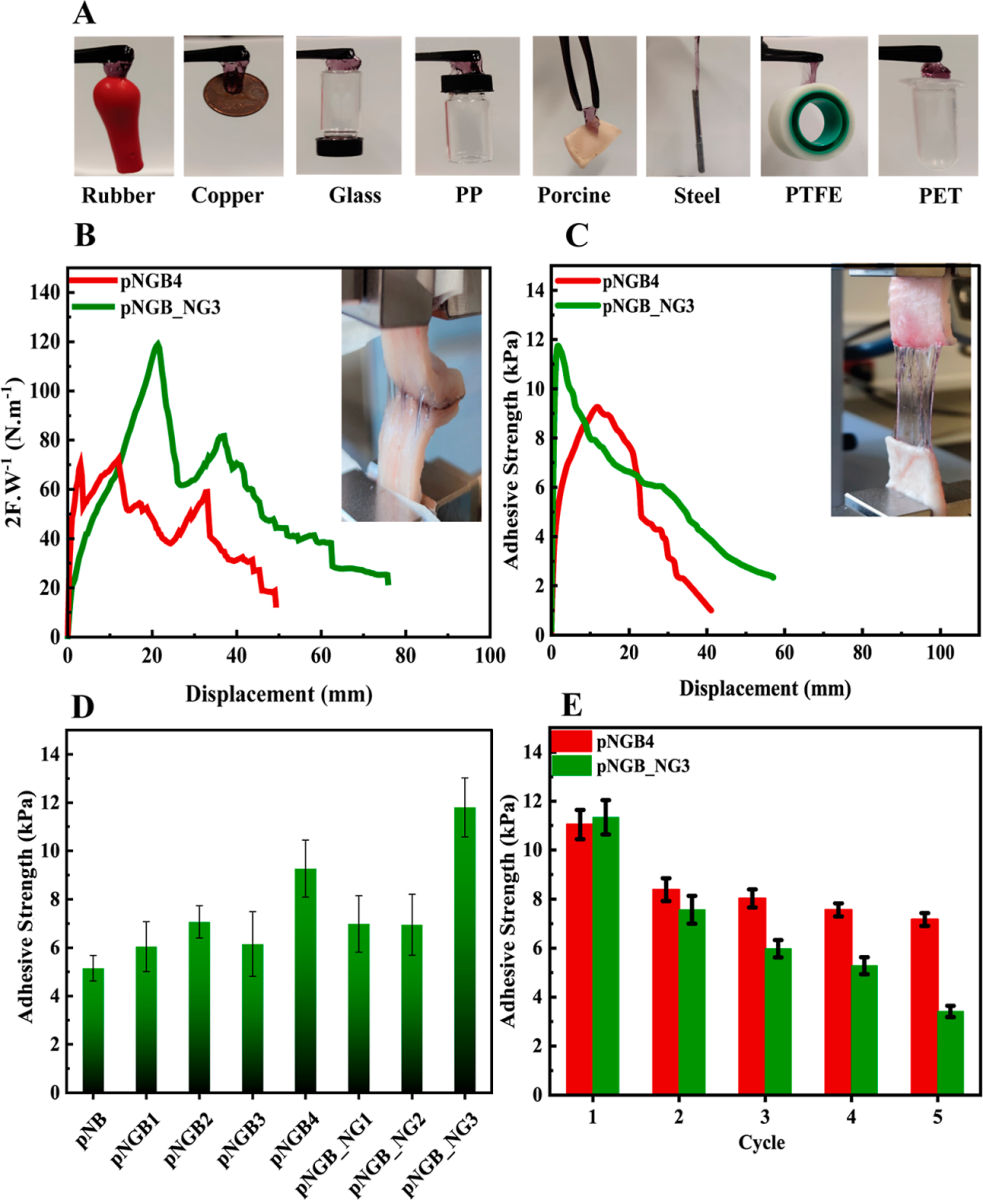
Adhesion of various surfaces to the prepared hydrogels (A), adhesion of pNGB_NG3 and pNGB4 in the T-peel test (B) and in lap-shear test results versus displacement (C). Adhesive strength of all the prepared samples (D) and cyclic adhesive strength results for pNGB_NG3 and pNGB4 (the mean values for three replicate samples ± standard deviation are shown) (E) in the lap-shear test.

We tested several materials to assess the adhesion properties of the prepared gel. The excellent adhesion of the hydrogel to various substrates, including rubber, copper, glass, polypropylene (PP), porcine tissue, steel, polytetrafluoroethylene (PTFE), and polyethylene terephthalate (PET), is illustrated in Figure 7A. The adhesion properties of the prepared hydrogels were further evaluated using T-peel, lap-shear, and cyclic adhesion tests.

During the T-peel test, the peeling force (*F*_p_) was measured while separating the hydrogel from two porcine tissues at a 180° angle. As can be seen in Figure 7B, the T-peel tests on porcine tissues demonstrated strong adhesion of the optimal hydrogel samples, with maximum peeling adhesion values of approximately 70 N·m^–1^ for pNGB4 and 120 N·m^–1^ for pNGB_NG3. The interfacial toughness values of pNGB_NG3 and pNGB4 were calculated and were approximately 22.5 and 25.3 J·m^–2^, respectively. The results of interfacial toughness are within satisfactory range.^57^

The lap-shear tests were conducted to assess the adhesive strength of the hydrogels. Shear forces were applied to two porcine tissues bonded with the hydrogel, and adhesive strength values were calculated based on the maximum shear force and the overlapping area. Figure 7C presents the dependence of adhesive strength versus displacement for both optimal samples for comparison, and Figure 7D shows the adhesion strength of all the prepared hydrogels to porcine tissue in the lap-shear test. The results from both Figure 7C,D indicate that higher concentrations of the GNPs, BISS, and the NIPAm monomers led to increased adhesion, while the sample without GNPs showed the lowest adhesive strength.^58^ Notably, samples containing NG exhibited higher adhesion compared to those without NG. In particular, pNGB_NG3 showed the highest adhesive strength of 2.4 kPa. Figure 7E presents the cyclic adhesion tests. The results revealed that the adhesion of pNGB4 remained relatively stable over five cycles, with only a slight decrease in the adhesion percentage compared to pNGB_NG3.

The adhesion results indicated that the adhesion of hydrogels to porcine tissue was influenced by the composition of the hydrogel, particularly the concentrations of GNPs, BISS, and NIPAm monomers. According to the literature, the presence of amide, amine, and carboxylic acid groups facilitated increased electrostatic interactions, hydrogen bonding, and chemical interactions with carboxyl and other functional groups on tissue proteins. The disulfide bonds in BISS can form covalent links with thiol groups present in proteins on the tissue surface. Additionally, the electrostatic interactions between negatively charged GNPs and positively charged regions on tissue surfaces, as well as hydrogen bonds and other noncovalent interactions, can contribute enhancement of adhesiveness.^59^ Furthermore, the presence of NG also enhanced the adhesion properties (through additional molecules of NIPAm and BISS). The stability of adhesion over multiple cycles indicated the suitability of these hydrogels for practical applications requiring repeated adhesion.

### 3.8 DOX Release

**Figure 8:**
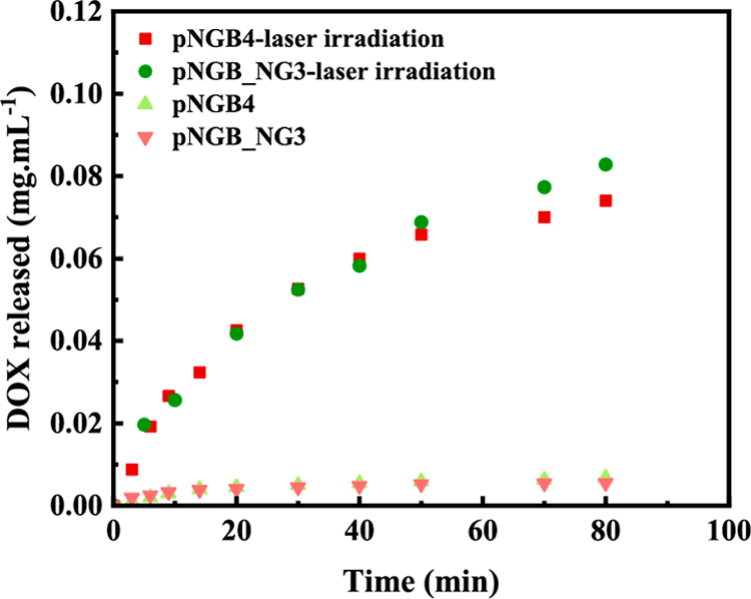
DOX released from optimal samples over time of irradiation and over time without irradiation. The test was done on three replicate samples, and the mean values are depicted in the graph.

DOX was selected as the model anticancer drug to evaluate its release from the hydrogel matrix. Biocompatibility testing is essential for potential clinical applications. The investigated hydrogel consists of components such as NIPAm-based hydrogels and gold nanoparticles, whose biocompatibility has already been confirmed in previous studies.^60,61^However, to ensure safety, further testing, such as cell viability assays should be conducted. NIR irradiation was utilized to induce drug release from both hydrogel formulations (pNGB4 and pNGB_NG3). The DOX with 48% DLC release as a function of time is illustrated in Figure 8. Initially, without irradiation, a very limited drug release was observed from both hydrogel formulations. However, upon laser irradiation, a significant increase in DOX release was observed over time. With continuous irradiation, the concentration of DOX in the buffer increased, reaching 0.07 and 0.08 mg·mL^–1^ for pNGB4 and pNGB_NG3, respectively, after 80 min. This study demonstrated that the laser irradiation triggered the release of DOX from the hydrogel matrix, with higher amounts released in the presence of NG. As mentioned previously, the introduction of NG into the hydrogel reduced its mechanical rigidity by increasing the intermolecular spacing between polymer chains and decreasing the number of cross-linking points between them. This led to higher amounts of DOX being released.

## 4 Conclusions

In conclusion, our study confirmed that the incorporation of *N,N′-*bis(acryloyl)cystine (BISS)-modified gold nanoparticles (GNPs) into pNIPAm hydrogels enhanced various properties, notably improving mechanical strength, energy dissipation, adhesion, and drug release. The integration of the photothermal properties of the hydrogels with laser irradiation facilitated rapid and nearly complete healing after damage. Furthermore, the introduction of p(NIPAm-BISS) nanogels (NG), synthesized from NIPAm and BISS, further augmented drug release capabilities and adhesion properties. These advancements address the demand from biomedicine for multifunctional materials suitable for applications in tissue engineering, drug delivery, and wound healing.

## Supporting Information Available

Tensile stress–strain curves of the hydrogels without GNPs (pure hydrogels), intensity of hydrodynamic diameter of the p(NIPA-BISS) nanogel measured at 37 °C, TEM images of the p(NIPA-BISS) nanogel (NG) and the size distribution of the nanogel particles, TEM images of the hydrogel and EDS-HAADF elemental mapping of Au, sulfur, and carbon in the hydrogel, and TEM images and EDS-HAADF elemental mapping of Au and sulfur in GNPs and BISS-modified GNPs (PDF)
