# Supplementary material for: Rapid Photoinduced Self-Healing, Controllable Drug Release, Skin Adhesion Ability, and Mechanical Stability of Hydrogels Incorporating Linker-Modified Gold Nanoparticles and Nanogels
Source: ACS Appl Mater Interfaces. 2024 Oct 8;16(42):57659–71. doi: 10.1021/acsami.4c11908 (PMC11503619; doi:10.1021/acsami.4c11908)
Supplement: Supplementary file 1 — am4c11908_si_001.pdf [file am4c11908_si_001.pdf]

Supporting Information

Rapid Photoinduced Self-Healing, Controllable Drug Release, Skin Adhesion Ability, and Mechanical Stability of Hydrogels Incorporating Linker-Modified Gold Nanoparticles and Nanogels

Samaneh Khodami[a,b], Mosayeb Gharakhloo[a,b], Serife Dagdelen[a], Piotr Fita[c], Jan Romanski[b], Marcin Karbarz[a,b], Zbigniew Stojek[b*], and Marcin Mackiewicz[a*]

[a] Biological and Chemical Research Center, University of Warsaw, Zwirki i Wigury 101, 02-089 Warsaw, Poland.

[b] Faculty of Chemistry, University of Warsaw, Pasteura 1, 02-093 Warsaw, Poland.

[c] Institute of Experimental Physics, Faculty of Physics, University of Warsaw, Pasteura 5, 02-093 Warsaw, Poland.

* Corresponding authors:

E-mail addresses: mmackiewicz@chem.uw.edu.pl and stojek@chem.uw.edu.pl

1. Pure hydrogels were evaluated by the standard tensile test to compare with pNGB4 and pNGB-NG3 mechanical results (Figure S1). The concentrations of the NIPAm monomer and BISS were set at 1.4 M and 0.9 mM, respectively. Figure S1's green curve depicts the hydrogel with NG included, while the red curve depicts the hydrogel without NG. Due to the Au-sulfur interaction, these data clearly demonstrated increasing strength after adding GNPs to the hydrogel structure.

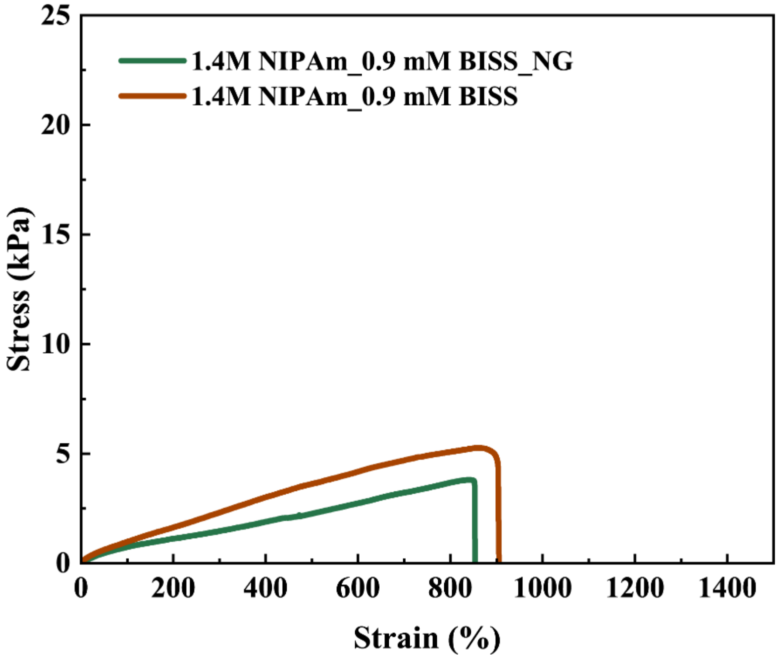

Figure S1. The stress-strain curves of the hydrogels without GNPs (pure hydrogels).

2. TEM image and DLS graph for the p(NIPAm-BISS) nanogel (NG) are placed in Figures S2 and S3. The mean hydrodynamic diameter of NG measured at 37°C equaled 48 ± 15 nm by DLS, whereas at 60°C it even reached to 20 ± 10 nm (DLS part). The TEM micrographs indicated that the dried gel was predominantly spherical, with an average diameter of approximately 26 ± 6 nm.

   The reason for the observed variation in particle sizes between TEM and DLS measurements is the difference between the hydrated and dehydrated states of the NG during DLS analysis and TEM imaging. TEM provides the true physical size of the dried particles, which leads to reduced observed dimensions, while DLS measures the hydrodynamic diameter, including the solvent layer, producing higher size readings. [1]

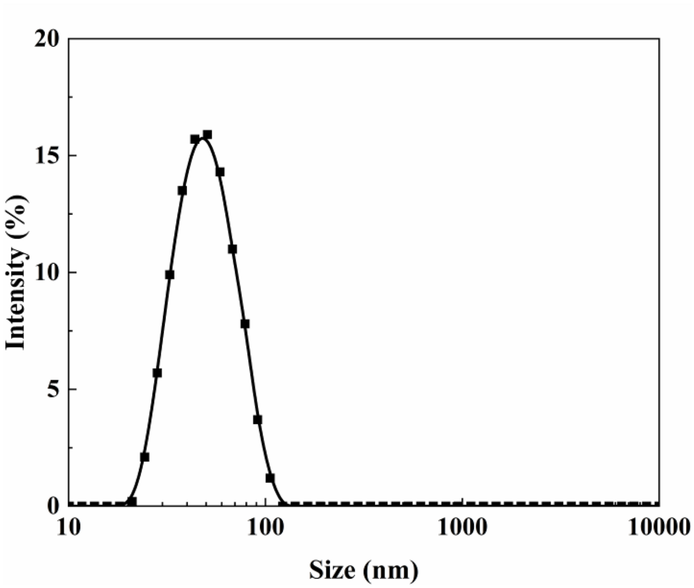

Figure S2. Intensity of hydrodynamic diameter of NG measured at 37 °C.

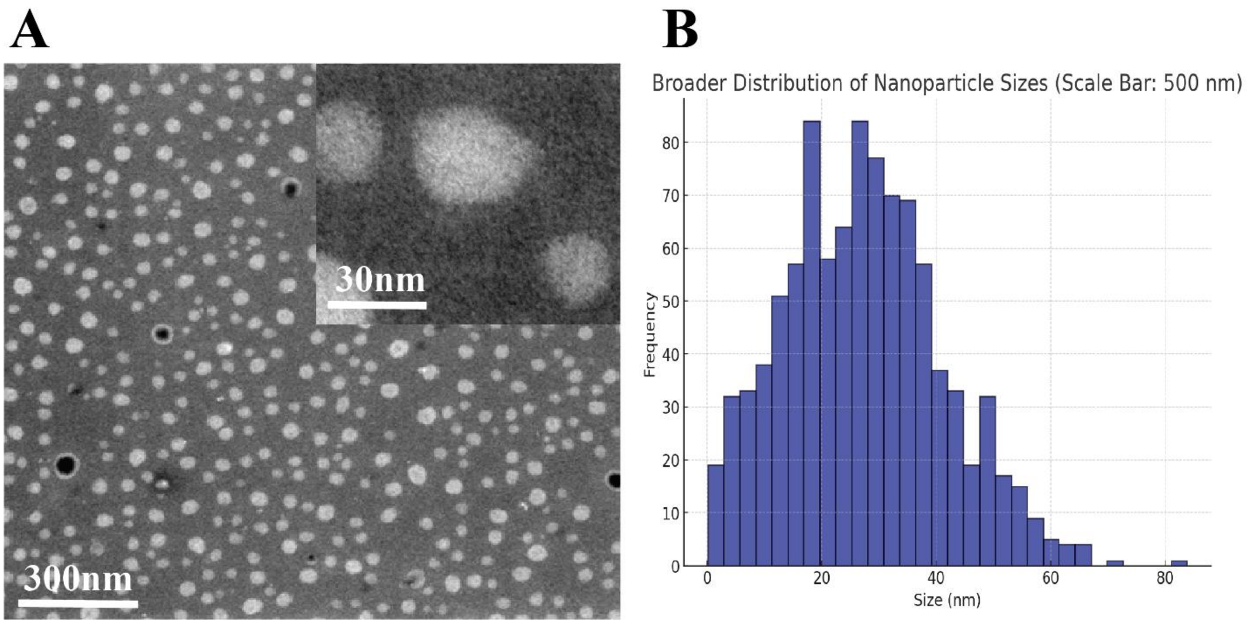

Figure S3. TEM images of NG (A) and the size distribution of NG particles (determined using Nano Measurer 1.2 software based on TEM images) (B).

3. The hydrogel surface of pNGB4 and GNPs and BISS-modified GNPs were characterized by high angle annular dark field scanning transmission electron microscopy (HAADF-STEM), and energy-dispersive X-ray spectrometry (HAADF-EDS) mapping showing the elemental distribution of gold, sulfur, and carbon.

Figure S4 showed good GNPs and BISS distribution in the hydrogel by EDS mapping of Au and sulfur through the hydrogel matrix and Figure S5 showed successful modification of GNPs with BISS by showing distribution of sulfur-covered GNPs.

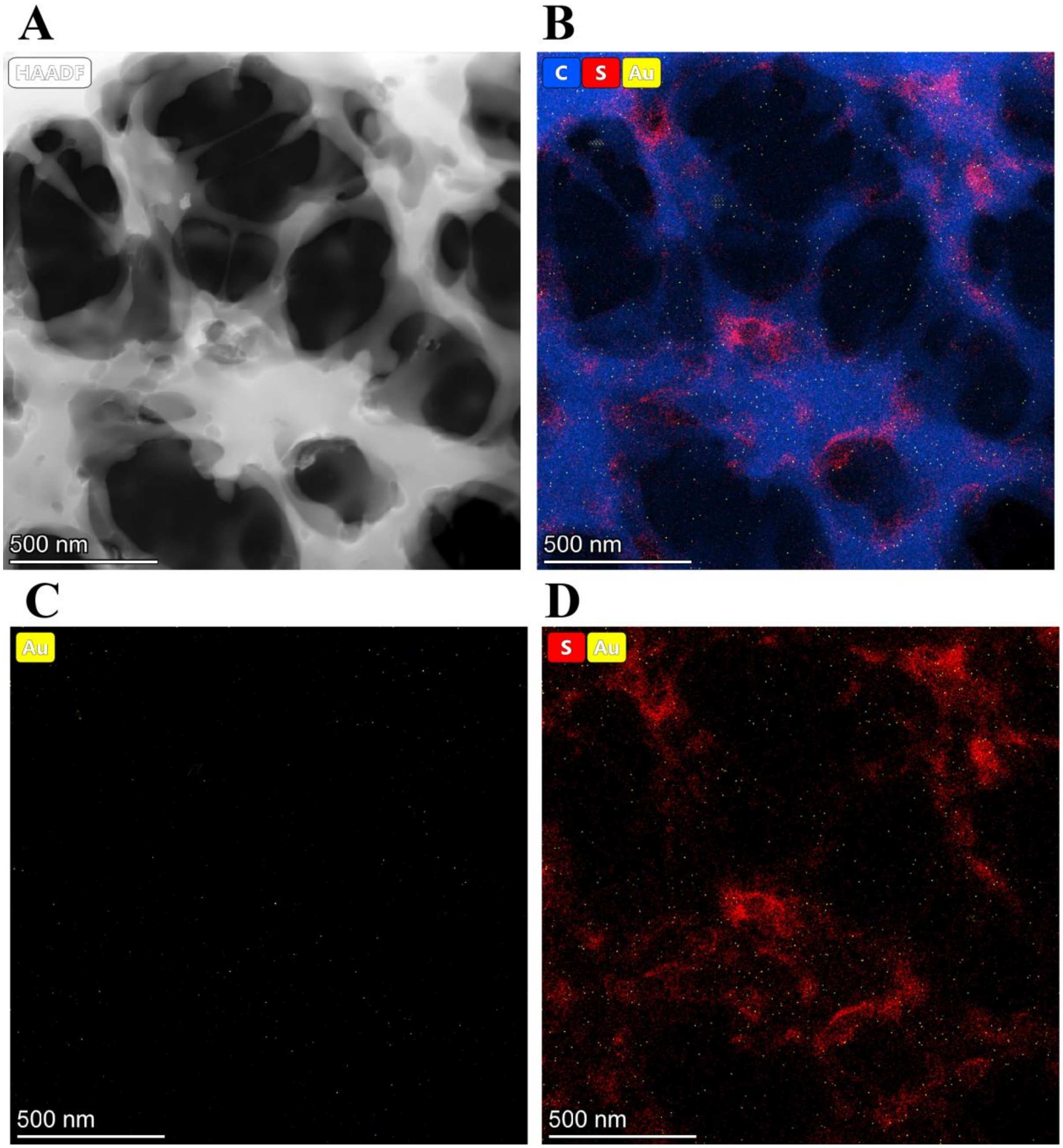

Figure S4. HAADF-STEM image of pNGB4 hydrogel (A), EDS-HAADF elemental mapping of gold, sulfur, and carbon (B), gold (C), and sulfur-covered gold (D).

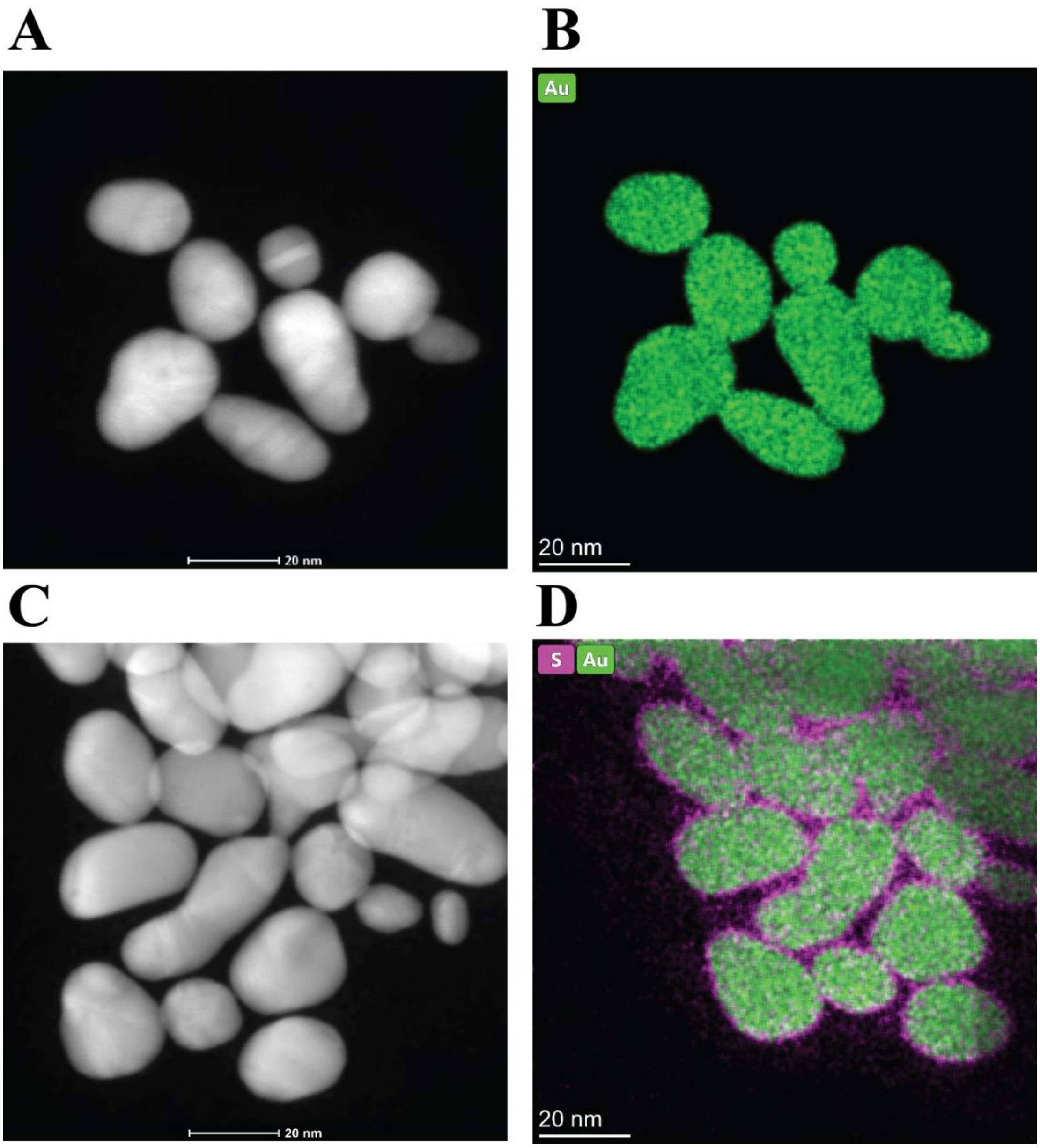

Figure S5. HAADF-STEM image of GNPs (A), EDS-HAADF elemental mapping of gold (B). HAADF-STEM image of the BISS modified-GNPs (C), and EDS-HAADF elemental mapping of gold and sulfur in BISS modified-GNPs (D).

1. Berne, Bruce J.; Robert Pecora. Dynamic Light Scattering: With Applications to Chemistry, Biology, and Physics. *Courier Corporation*, **2000**.
